# Mental health and sociodemographic characteristics among Icelanders, data from a cross-sectional study in Iceland

**DOI:** 10.1186/s12888-022-04504-y

**Published:** 2023-01-12

**Authors:** Svala Sigurðardóttir, Thor Aspelund, Dóra G. Guðmundsdóttir, Lone Fjorback, Hannes Hrafnkelsson, Ingunn Hansdóttir, Lise Juul

**Affiliations:** 1 Centre of Public Health Sciences, Faculty of Medicine, University of Iceland, Sturlugata 8, 101 Reykjavik, Iceland; 2 Department of Clinical Medicine, Danish Center for Mindfulness, Aarhus University, Aarhus, Denmark; 3 Directorate of Health, Reykjavik, Iceland; 4 Primary Care of the Capital Area, Reykjavík, Iceland

**Keywords:** Mental health, Depression, Anxiety, Stress, Sociodemographic factors, DASS-21, Gender, Financial difficulties, Prevalence, Cross-sectional

## Abstract

**Background:**

Mental health challenges are on the rise worldwide. In Iceland, little is known about the sociodemographic factors associated with poor mental health. This study aimed to investigate symptoms of depression, anxiety, stress, and psychiatric medication for mental disorders in a nationally representative sample in Iceland and to explore its associations with sociodemographic factors.

**Methods:**

This Icelandic cross-sectional study ‘Health and Wellbeing of Icelanders’ was conducted in 2017 and included 9,887 randomly chosen adults. Participants’ depression, anxiety, and stress levels were measured with the Depression Anxiety and Stress scale-21(DASS-21) and the association with sociodemographic factors and prescribed psychiatric medication was assessed in a multinominal logistic regression analysis.

**Results:**

The youngest age group (18 to 29 years old) had the poorest mental health. Males had a higher risk of medium and high depression scores than females, RRR 1.23 (95% CI 1.06–1.44) and RRR 1.71 (95% CI 1.25–2.33) when adjusted for sociodemographic factors (age, sex, education, marital status, financial status, living area, employment) and use of psychiatric medication. Participants with the most considerable financial difficulties had the highest risk of high scores on depression RRR 11.19 (95% CI 5.8—21.57), anxiety RRR 12.35 (95% CI 5.62—27.14) and stress RRR 11.55 (95% CI 4.75—28.04) when compared to those that do not.

**Conclusions:**

The youngest participants and those with the most extensive financial difficulties had the highest depression, anxiety, and stress scores. Males scored higher than females on depression. There was a trend towards worse mental health with lower sociodemographic status. Higher education, living with someone, and financial security were associated with better mental health. These results implicate the importance of government actions to counteract social inequalities in the Icelandic nation.

**Supplementary Information:**

The online version contains supplementary material available at 10.1186/s12888-022-04504-y.

## Background

Mental disorders affect numerous individuals in their lifetime [1], and although they’re associated with premature mortality [2], they remain an underestimated aspect of public health [3]. Depressive disorders have been one of the three leading causes of disability for almost three decades. From 1990 to 2007, the number of years lived with disability (YLDs) due to depression and anxiety rose globally by over 30% and continued to rise during the past decade. From 2007 to 2017, YLDs due to depression rose by 14.3% and 12.8% due to anxiety [4]. This trend is likely to continue, even escalate, in the coming years.

In the pre-pandemic European Union, over one-third of the population (38.2%) experienced symptoms of mental disorders annually, with anxiety (14.0%) and major depression (6.9%) being most prevalent [5].

Comorbidity of anxiety and depression is common [6] and often related to stress, an independent risk factor for morbidity and mortality [7].

A new Danish population study report from 2021 documented a 4%-point increase from 2017 (25.1%—29.1%) in the prevalence of high stress measured by the Perceived Stress Scale and a 4.2%-point increase (13.2%—17.4%) in self-reported poor mental health [8]. The youngest age groups (16–24 years old) scored highest and showed the most significant increase in high perceived stress level from 2017, men 23.4% to 31.2% and women 40.5% to 52.3% [8]. This increase in perceived stress is important to recognize because increased stress levels can affect both mortality [9] and the use of primary healthcare and mental health-related services [9].

The same trend was reported for the youngest age group and poor self-reported mental health, for both sexes. The increase for the youngest women was 10.6% and 8.2% for the youngest men [8].

Many studies show that mental disorders and stress are more prevalent among women than men [1, 8, 10–12].

Low income and debts are associated with mental disorders [13, 14] and stress [8, 15] and studies have suggested that high chronic stress levels are more common in people with low socioeconomic status [11].

Adverse effects of unemployment on mental health are apparent, and unemployment is associated with higher perceived stress [9] and mental disorders [14, 16].

Age is associated with mental health [8], older age groups often show lower depression, anxiety, and stress symptoms, however, there is a proneness for a slight increase in the oldest age group [8].

Mental disorders and poor self-reported mental health are also associated with marital status [8, 14]. A Danish study found that divorcees had greater odds of having depression and anxiety than those married and unmarried or divorced people had lower mental well-being than those who were married [14]. Perceived stress has also been shown to be higher for those living alone [8, 9].

Low educational levels increase the odds of depression and anxiety [14] and it is associated with higher stress scores and lower mental health [8].

Few studies have assessed the prevalence of stress and mental disorders in Iceland. The most recent one reported a 49.8% lifetime prevalence of any ICD-10 mental disorders among 34–74 years old Icelanders living in the Great Reykjavik area [17]. A lifetime prevalence criteria for any anxiety disorder were met in 14.4% participants and for any mood disorder in 13.0% [17]. The 1-year prevalence for anxiety disorders was 5.5% and for mood disorders 2.6%. These data are over a decade old and important to re-evaluate, not least in light of the current global increase of mental health challenges [1, 18]. This paper provides insight into the current mental health status of the Icelandic adult population and further explains the factors affecting it.

Hence, mental health is an essential aspect of public health, an important index that must be monitored nationally with studies of associated sociodemographic factors.

### Aims

The present study aimed to investigate self-reported depression, anxiety, and stress symptoms and the use of psychiatric medication amongst Icelanders older than 18 in 2017 and to assess the associations of mental health challenges with sociodemographic factors.

We hypothesized – based on current national and international data – that the youngest age groups particularly and women in general would have the highest prevalence of mental health challenges, and that lower sociodemographic status could have negative impact on these symptoms.

## Methods

### Study design and setting

This cross-sectional study is based on data from the national health survey “Health and Wellbeing of Icelanders” gathered from October 2017 until February 2018 by the Directorate of Health in Iceland19.

### Participants

**Fig. 1:**
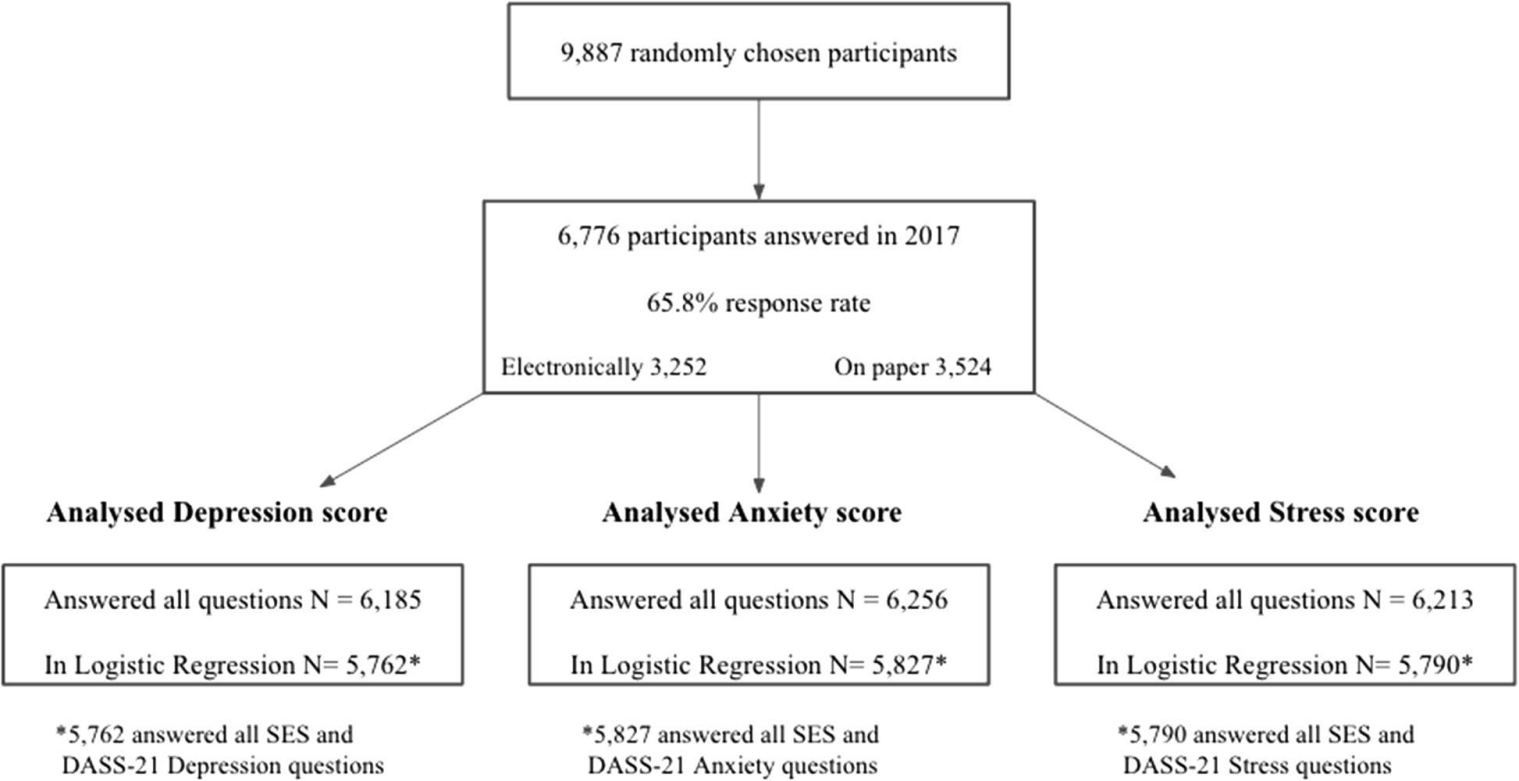
Flow diagram of participants in the study and their response rate on DASS-21 and other questions (Health and wellbeing of Icelanders 2017)

The data included 6,776 of the 9,887 eligible participants, Fig. 1 demonstrates their response rate.

The Icelandic Directorate of Health attained a random population sample from Statistics Iceland, including all Icelanders older than 18 living in Iceland at the time. The response rate was 68.5% [19],a total of 6,776 respondents, and the sample was stratified to ensure sufficient participation of all age groups and from all geographical areas. We only analysed those who completed the Depression Anxiety and Stress – 21 scale (DASS-21-scale) questionnaire.

### Outcome measures and co-variables

The primary outcomes of interest were scores on the shorter version of the DASS [20] scale called DASS-21 [21], and includes 21 of the 42 questions of the DASS scale. There are seven questions for each part (depression, anxiety, and stress) designed to measure the severity of symptoms common to depression, anxiety, and stress during the previous week. Participants were asked to rate the extent to which they had experienced each symptom on a 4-point Likert scale with possible scores for each answer ranging from 0 (did not apply to me at all) to 3 points (applied to me very much/most of the time), the higher a score, the worse the symptoms. The total scores for each subgroup are divided into five categories: normal (0–9 for depression, 0–7 for anxiety and 0–14 points for stress), mild (10–13 for depression, 8–9 for anxiety and 15–18 points for stress), moderate (14–20 for depression, 10–14 for anxiety and 19–25 points for stress) severe (21–27 for depression, 15–19 for anxiety and 26–33 points for stress) and extremely severe (all scores above). The score attained from the DASS-21 are doubled to yield a score, comparable to the DASS scale.

The DASS-21 scale is reliable and has been validated in clinical [20] and non-clinical samples [22]. The scale has been validated in Icelandic and is deemed to have the same correlation between DASS and DASS-21 as in other languages and sufficient validity (Ingimarsson, B. The psychometric testimonials on the DASS Self-Assessment Scale. Depression, anxiety and stress. Unpublished cand. psych. Dissertation). 

### Co-variables

*Sex* was defined as male or female. *Age* was obtained by participants’ year of birth as documented in the survey and divided into 10-year ranges with the last age range, 70 + years, being open ended. *Education* was the level of attained education, divided into three: basic, middle, and university, with basic indicating compulsory education from 6 to 16 years of age, middle representing secondary school and technical or vocational training, and university representing degrees starting with the bachelor level.

*Marital status* contained five categories: married/cohabiting, dating, divorced, single, and widowed. *Financial difficulty* was assessed by the question: “How easy or difficult has it been for you and your family to make ends meet over the past 12 months?”. Possible answers: very easy, rather easy, neither, difficult, and very difficult. *Residency* was defined as either urban or rural, with the former group living in the greater Reykjavik area.

*Unemployment* was dichotomized into unemployed or not. Information on *current and previous medical treatment for mental disorders* was obtained from three questions “Have you taken prescribed medication for depression/anxiety/other mental disorders?”, with possible answers (a) “yes, in the past two weeks” (currently), (b) “yes, more than two weeks ago” (previously) and (c) “no, never” (never).

### Statistical methods

Data were analysed using StataIC (version 15). We calculated the scores on DASS-21 for all participants and divided into three groups: Normal, mild/moderate (called medium), or severe/extremely severe (called high) scores for each subgroup (depression, anxiety, and stress).

Scoring ranges are presented as follow: normal (0–9 for depression, 0–7 for anxiety and 0–14 points for stress), medium (10–20 for depression, 8–14 for anxiety and 15–25 points for stress) and high (> 20 for depression, > 14 for anxiety and > 25 points for stress).

(The distribution of the full DASS-21 scores by severity groups and by gender are displayed in Additional file 1, supplementary tables 4 and 5).

The data were analysed by multinomial logistic regression adjusting for co-variates: age, sex, education, marital status, financial difficulty, living area, unemployment, and current and previous use of psychiatric medication for mental disorders.

This approach allowed us to estimate the relative risk ratio (RRR) within each group of participants, comparing the risk of scoring medium or high to scoring normal and controlling for the confounding variables.

To study effect modification by medication, and sex we performed sub-group regression analysis and evaluated the significance of interaction effects in the regression analysis, adjusting for other covariates.

Multinomial logistic regression was performed to look at dissimilarities in education between the sexes, since studies have demonstrated that men with lower educational levels seek less medical help for mental health problems [23] than women. A likelihood-radio test was performed additionally to see if antidepressant medication intake was an effect modifier of the gender differences in depression scores. (Additional file 1, supplementary tables 1,2 and 3).

## Results

A total of 54.6% of the participants were female, and 70.7% of the participants were more than 49 years old; most of them were married or cohabiting (71.6%) and had middle-level or high educational attainment (39.6% and 29.4%). 12.4% reported financial difficulties, approximately half of the participants lived in urban settings (48.3%) and 2.4% were unemployed. A vast majority of the participants had never taken medication for anxiety (83.0%), depression (88.2%) or other mental disorders (94.6%).

### Self-reported mental health

Participants’ mean DASS-21 score by sociodemographic factors is presented in Table 1.

**Table 1:** Average DASS-21 score of all participants by sociodemographic factors (Health and wellbeing of Icelanders 2017)

**DASS-21 score**			**Depression score**		**Anxiety score**		**Stress score**	
	N	%	Mean	SD	Mean	SD	Mean	SD
**Age group**								
18–29	371	5.5	8.1	9.1	4.3	5.8	10.4	8.1
30–39	650	9.6	6.3	7.6	3.3	4.7	9.3	7.5
40–49	962	14.2	5.3	6.7	2.4	4.0	7.8	6.6
50–59	1247	18.5	5.3	7.1	2.6	4.5	7.0	6.6
60–69	1535	22.7	4.8	6.4	2.7	4.3	5.7	6.0
70 >	1990	29.5	5.3	6.4	3.0	4.3	4.7	5.6
**Gender**								
Female	3684	54.6	5.6	7.0	3.2	4.7	6.9	6.8
Male	3063	45.4	5.2	6.7	2.5	4.0	6.3	6.4
**Education**								
Basic	2043	31.0	6.4	7.6	3.7	5.3	6.7	6.8
Middle	2600	39.4	5.1	6.6	2.7	4.1	6.3	6.5
University	1949	29.6	4.9	6.4	2.3	3.9	7.1	6.6
**Marital status**								
Married/cohabiting	4802	71.6	4.8	6.4	2.6	4.2	6.5	6.5
Dating	151	2.3	7.1	7.6	4.5	6.5	9.3	7.6
Divorced	279	4.2	5.3	6.3	2.9	4.5	7.0	6.7
Single	930	13.9	8.1	8.7	3.7	5.1	7.6	7.1
Widowed	545	8.1	5.8	6.8	3.4	4.8	4.6	5.5
**How easy making ends meet**								
Very easy	1990	30.4	4.1	5.6	2.0	3.2	5.3	5.6
Rather easy	1979	30.3	4.8	6.2	2.5	4.0	6.1	6.2
Neither nor	1764	27.0	6.0	7.3	3.2	4.8	7.3	6.8
Rather difficult	670	10.3	8.3	8.2	4.7	5.7	9.4	7.8
Very difficult	134	2.1	14.5	10.9	8.1	8.0	13.5	9.5
**Urban/rural**								
Urban	3243	48.3	5.2	6.6	2.7	4.2	6.4	6.5
Rural	3469	51.7	5.7	7.2	3.0	4.6	6.9	6.8
**Employment**								
Unemployed	154	2.4	10.0	10.0	5.0	6.0	9.5	8.7
Not unemployed	6361	97.6	5.3	6.8	2.8	4.4	6.6	6.6
**Taking medication for anxiety**								
Currently	561	8.5	12.4	10.3	7.4	7.5	12.4	8.6
Previously	563	8.5	8.3	7.9	4.7	5.2	9.8	7.0
Never	5499	83.0	4.4	5.8	2.2	3.5	5.7	5.9
**Taking medication for depression**								
Currently	420	6.3	14.6	10.8	7.6	7.8	12.9	9.0
Previously	362	5.5	10.1	7.9	5.0	5.7	11.0	7.6
Never	5843	88.2	4.5	5.8	2.4	3.7	5.9	6.0
**Taking medication for other mental**								
Currently	162	2.5	14.2	11.3	9.1	8.7	14.4	9.4
Previously	195	3.0	9.7	9.3	5.0	6.1	11.0	8.1
Never	6251	94.6	5.0	6.4	2.6	4.0	6.3	6.3

The youngest age group had the highest average DASS-21 scores. There were minor differences in average depression (females 5.6, males 5.2) anxiety (females 3.2, males 2.5) and stress scores (females 6.9, males 6.3) between sexes. Those with lowest educational level, had highest average depression and anxiety scores, but those with the highest education, had highest average stress score. Single participants had the highest average depression score but those dating had highest anxiety and stress scores. The highest average depression score was seen for those currently taking medication for depression (14.6). The highest average anxiety and stress score were seen those currently taking medication for other mental diseases. Participants currently or previously using psychiatric medicine, score considerably higher on DASS-21, than those that had never taken these medications. Higher scores are seen for the unemployed, likewise, participants with financial difficulties. The distribution of DASS-21 scores by levels of normal, medium and high are presented in Table 2.

**Table 2:** Distribution of DASS-21 scores by sociodemographic factors (Health and wellbeing of Icelanders 2017)

	**DASS-21 Depression score**						**DASS-21 Anxiety score**						**DASS-21 Stress score**					
	Normal		Medium		High		Normal		Medium		High		Normal		Medium		High	
	< 10		10 to 20		> 20		< 8		8 to 14		> 14		< 15		15 to 25		> 25	
	N	%	N	%	N	%	N	%	N	%	N	%	N	%	N	%	N	%
**Age group**																		
18–29	242	68.8	74	21.0	36	10.2	288	80.5	47	13.1	23	6.4	270	76.7	59	16.8	23	6.5
30–39	471	75.4	116	18.6	38	6.1	527	84.5	74	11.9	23	3.7	520	82.2	85	13.4	28	4.4
40–49	743	79.4	153	16.4	40	4.3	842	89.8	75	8.0	21	2.2	806	86.7	106	11.4	18	1.9
50–59	941	79.2	188	15.8	59	5.0	1060	88.2	108	9.0	34	2.8	1065	89.0	109	9.1	23	1.9
60–69	1166	81.7	216	15.1	45	3.2	1285	89.1	120	8.3	37	2.6	1326	92.9	86	6.0	16	1.1
70 >	1326	80.0	277	16.7	54	3.3	1476	87.3	178	10.5	37	2.2	1588	95.0	65	3.9	19	1.1
**Gender**																		
Female	2638	78.5	565	16.8	156	4.6	2908	85.3	380	11.2	120	3.5	2997	89.1	287	8.5	78	2.3
Male	2247	79.8	454	16.1	115	4.1	2562	90.3	221	7.8	54	1.9	2570	90.5	221	7.8	49	1.7
**Education**																		
Basic	1305	73.7	359	20.3	108	6.1	1491	82.6	236	13.1	78	4.3	1585	89.0	155	8.7	42	2.4
Middle	1977	80.9	375	15.3	93	3.8	2208	89.2	214	8.6	54	2.2	2230	90.5	189	7.7	46	1.9
University	1558	81.7	280	14.7	69	3.6	1728	90.5	144	7.5	38	2.0	1703	89.4	163	8.6	39	2.1
**Marital status**																		
Married/ cohabiting	3668	82.2	638	14.3	155	3.5	4010	89.0	389	8.6	105	2.3	4046	90.3	348	7.8	85	1.9
Dating	97	69.3	32	22.9	11	7.9	111	78.2	20	14.1	11	7.8	117	81.8	18	12.6	8	5.6
Divorced	194	74.9	59	22.8	6	2.3	231	88.5	22	8.4	8	3.1	229	88.1	27	10.4	4	1.5
Single	569	66.9	201	23.7	80	9.4	714	82.8	115	13.3	33	3.8	729	85.9	97	11.4	23	2.7
Widowed	345	76.8	87	19.4	17	3.8	391	85.0	53	11.5	16	3.5	433	95.4	16	3.5	5	1.1
**How easy making ends meet**																		
Very easy	1610	85.5	233	12.4	40	2.1	1782	93.6	101	5.3	20	1.1	1774	94.1	98	5.2	14	0.7
Rather easy	1526	82.4	271	14.6	54	2.9	1665	89.2	165	8.8	37	2.0	1699	91.3	133	7.2	29	1.6
Neither nor	1243	76.9	289	17.9	85	5.3	1406	85.5	182	11.1	57	3.5	1442	88.5	148	9.1	40	2.5
Rather difficult	387	63.1	169	27.6	57	9.3	469	75.5	118	19.0	34	5.5	499	80.7	89	14.4	30	4.9
Very difficult	48	38.4	43	34.4	34	27.2	74	59.7	25	20.2	25	20.2	73	59.4	36	29.3	14	11.4
**Urban/rural**																		
Urban	2405	80.0	496	16.5	107	3.6	2667	88.1	286	9.5	74	2.4	2711	90.1	246	8.2	51	1.7
Rural	2477	78.3	522	16.5	165	5.2	2803	87.1	315	9.8	99	3.1	2853	89.4	262	8.2	76	2.4
**Employment**																		
Unemployed	87	60.8	33	23.1	23	16.1	105	72.9	26	18.1	13	9.0	112	79.4	21	14.9	8	5.7
Not unemployed	4710	79.8	953	16.1	243	4.1	5254	88.0	560	9.4	157	2.6	5341	90.0	477	8.0	119	2.0
**Taking medication for anxiety**																		
Currently	241	46.6	167	32.3	109	21.1	311	59.9	128	24.7	80	15.4	352	68.5	112	21.8	50	9.7
Previously	329	62.8	153	29.2	42	8.0	397	75.2	105	19.9	26	4.9	424	80.0	90	17.0	16	3.0
Never	4256	84.2	686	13.6	115	2.3	4698	91.7	359	7.0	66	1.3	4724	92.9	301	5.9	59	1.2
**Taking medication for depression**																		
Currently	145	37.0	137	35.0	110	28.1	240	60.5	91	22.9	66	16.6	255	64.7	92	23.4	47	11.9
Previously	186	55.5	109	32.5	40	11.9	248	73.8	67	19.9	21	6.3	260	76.3	61	17.9	20	5.9
Never	4496	83.7	761	14.2	117	2.2	4923	90.5	432	7.9	85	1.6	4986	92.4	351	6.5	58	1.1
**Taking medication for other mental**																		
Currently	61	40.4	51	33.8	39	25.8	84	54.6	30	19.5	40	26.0	85	55.9	45	29.6	22	14.5
Previously	101	57.7	49	28.0	25	14.3	132	73.7	36	20.1	11	6.2	134	74.4	33	18.3	13	7.2
Never	4664	81.0	904	15.7	191	3.3	5185	89.1	519	8.9	118	2.0	5272	91.2	418	7.2	89	1.5

In the youngest age group, 21.0% score medium and 10.2% score high on depression, 13.1% score medium and 6.4% high on anxiety and 16.8% score medium and 6.5% high on stress. Small differences in depression and stress scores were seen between the genders, the largest difference was for medium and high anxiety scores (3.4 and 1.6%-point difference).

The highest proportions of high anxiety and stress scores (26% and 14.5%) were seen for those currently taking medication for other mental diseases and highest proportion of high depression scores (28.1%) was seen for those currently taking medication for depression.

### Sociodemographic factors associated with mental health

The adjusted Relative risk ratio (RRR) of scoring medium and high on DASS-21, compared to scoring normal, within each group is displayed in Table 3.

**Table 3:** The relative risk ratio of medium and high DASS-21 scores compared to normal scores (Health and wellbeing of Icelanders 2017)

	*n* = 5762				*n* = 5827				*n* = 5790			
	Medium VS Normal		High VS Normal		Medium VS Normal		High VS Normal		Medium VS Normal		High VS Normal	
	RRR	95%CI	RRR	95%CI	RRR	95%CI	RRR	95%CI	RRR	95%CI	RRR	95%CI
Age group												
18–29	reference group		reference group		reference group		reference group		reference group		reference group	
30–39	0.86	0.60–1.25	0.53	0.30–0.96	0.93	0.60–1.45	0.50	0.24–1.05	0.68	0.45–1.02	0.58	0.30–1.14
40–49	0.74	0.52–1.05	0.38	0.22–0.68	0.62	0.40–0.95	0.29	0.14–0.61	0.55	0.37–0.81	0.25	0.12–0.50
50–59	0.70	0.50–0.98	0.45	0.26–0.76	0.71	0.47–1.07	0.40	0.21–0.78	0.41	0.28–0.61	0.22	0.11–0.44
60–69	0.68	0.49–0.96	0.29	0.17–0.52	0.68	0.45–1.02	0.42	0.21–0.82	0.27	0.18–0.40	0.16	0.08–0.34
70 >	0.70	0.50–0.98	0.32	0.18–0.57	0.82	0.54–1.23	0.34	0.17–0.70	0.17	0.11–0.26	0.20	0.09–0.41
Gender												
Female	reference group		reference group		reference group		reference group		reference group		reference group	
Male	1.23	1.06–1.44	1.71	1.25–2.33	0.76	0.62–0.93	0.73	0.49–1.07	1.26	1.03–1.56	1.11	0.74–1.69
Education												
Basic	reference group		reference group		reference group		reference group		reference group		reference group	
Middle	0.70	0.58–0.84	0.70	0.49–0.99	0.73	0.58–0.91	0.61	0.40–0.92	0.83	0.64–1.07	0.95	0.59–1.53
University	0.68	0.55–0.84	0.72	0.49–1.07	0.59	0.45–0.77	0.48	0.30–0.79	0.82	0.62–1.08	0.91	0.54–1.55
Marital status												
Married/cohabiting	reference group		reference group		reference group		reference group		reference group		reference group	
Dating	1.61	1.02–2.52	1.48	0.64–3.41	1.59	0.93–2.70	1.95	0.84–4.52	0.93	0.52–1.67	1.66	0.70–3.95
Divorced	1.63	1.17–2.27	0.69	0.29–1.68	0.89	0.54–1.45	0.81	0.32–2.02	1.27	0.81–1.98	0.85	0.29–2.45
Single	1.57	1.27–1.92	2.06	1.46–2.92	1.09	0.84–1.41	0.74	0.46–1.20	0.97	0.74–1.27	0.68	0.40–1.15
Widowed	1.25	0.92–1.71	1.24	0.63–2.44	0.86	0.58–1.28	1.23	0.61–2.48	0.53	0.27–1.04	0.72	0.26–1.98
How easy making ends meet												
Very easy	reference group		reference group		reference group		reference group		reference group		reference group	
Rather easy	1.13	0.92–1.38	1.27	0.80–2.03	1.51	1.15–1.98	1.83	0.99–3.38	1.38	1.04–1.83	1.94	0.98–3.83
Neither nor	1.35	1.10–1.65	2.05	1.32–3.17	1.82	1.38–2.38	2.84	1.58–5.09	1.57	1.18–2.09	2.81	1.47–5.40
Rather difficult	2.35	1.83–3.02	3.91	2.41–6.33	3.20	2.34–4.38	4.03	2.10–7.74	2.28	1.63–3.19	4.74	2.36–9.52
Very difficult	3.37	2.08–5.44	11.19	5.80–21.57	3.23	1.87–5.58	12.35	5.62–27.14	5.06	3.01–8.52	11.55	4.75–28.04
Urban/rural												
Urban	reference group		reference group		reference group		reference group		reference group		reference group	
Rural	0.94	0.81–1.10	1.53	1.13–2.07	0.91	0.75–1.10	1.12	0.78–1.61	0.95	0.77–1.16	1.35	0.91–2.01
Employment												
Unemployed	reference group		reference group		reference group		reference group		reference group		reference group	
Notunemployed	0.75	0.47–1.19	0.42	0.22–0.79	0.57	0.35–0.94	0.58	0.26–1.30	0.82	0.46–1.47	0.66	0.28–1.58
Taking medication for anxiety												
Currently	reference group		reference group		reference group		reference group		reference group		reference group	
Previously	1.04	0.74–1.48	0.73	0.42–1.25	0.83	0.57–1.21	0.51	0.27–0.96	1.02	0.68–1.54	0.54	0.25–1.18
Never	0.52	0.39–0.70	0.30	0.19–0.48	0.31	0.22–0.43	0.19	0.11–0.32	0.39	0.27–0.55	0.42	0.22–0.78
Taking medication for depression												
Currently	reference group		reference group		reference group		reference group		reference group		reference group	
Previously	0.69	0.46–1.03	0.39	0.22–0.70	0.93	0.59–1.48	0.72	0.36–1.46	0.77	0.48–1.25	0.67	0.32–1.39
Never	0.34	0.25–0.47	0.13	0.08–0.21	0.65	0.45–0.93	0.42	0.24–0.73	0.62	0.42–0.92	0.21	0.11–0.39
Taking medication for other mental												
Currently	reference group		reference group		reference group		reference group		reference group		reference group	
Previously	0.68	0.38–1.21	0.76	0.36–1.61	0.77	0.41–1.47	0.25	0.11–0.59	0.55	0.30–1.01	0.63	0.27–1.50
Never	0.52	0.33–0.82	0.35	0.20–0.62	0.68	0.41–1.12	0.20	0.11–0.35	0.38	0.24–0.59	0.29	0.15–0.55

In these adjusted analyses, similar trends are seen in Table 2 with decreasing risk for medium depression with age, with 32% lower risk for 60- to 69-years-old compared to the youngest, 18–29 years old, RRR 0.68 (95% CI 0.49—0.98).

Likewise, a significantly lower risk for high depression was seen with increasing age, 47% lower for 29—39 years old compared to the youngest (18—29-years old) RRR 0.53 (95% CI 0.30—0.96); and 68% lower for 70 and older: RRR 0.32 (95% CI 0.18—0.57).

The risk of high anxiety and stress scores also decreased significantly with age, likewise for the medium stress symptoms, when all age groups above 39 years old were compared to the youngest age group.

The raw scores in Tables 1 and 2 demonstrated similar depression and stress scores for the sexes, but significant risk differences appeared after adjustments for sociodemographic factors (Table 3). Males have a 23% higher risk of medium depression score and 71% higher risk of high depression score than females, with RRR of 1.23; (95% CI 1.06—1.44) and 1.71; (95% CI 1.25—2.33). Males have a 26% higher risk of medium stress score as well, RRR 1.26; (95% CI 1.03—1.56), while they have a 24% lower risk for medium anxiety score, RRR 0.76; (95% CI 0.62—0.93).

The most significant risk differences are seen when comparing those who have financial problems with those that do not demonstrating over ten times larger risk for high depression, RRR 11.19; (95% CI 5.80 to 21.57), anxiety RRR 12.35; (95% CI 5.62 to 27.14), and stress RRR 11.55; (95% CI 4.75 to 28.94). Rural living was significantly associated with a higher risk of high depression than urban living and those employed had a statistically significantly lower risk of high depression score, RRR 0.42 and medium anxiety score RRR 0.57. Notably, participants taking medication for mental disorders had a much higher risk of medium and high depression, anxiety, and stress than those who had never taken medication for mental disorders. This was statistically significant for all groups except for medium anxiety (Table 3). The subgroup analysis we made to explain the differences between the sexes regarding depression score and education did not reveal any clear explanation. Still, psychiatric medication was an effect modifier, possibly influencing the difference between the sexes (Prob > chi2 = 0.03). (Additional file 1, Tables 1, 2 and 3).

## Discussion

The present study confirmed the hypothesis that the youngest participants have the highest prevalence of depression, anxiety, and stress, a well-known trend in the international literature [7–9]. Although the average depression scores between the sexes are very similar, males seemed to have higher depression and stress scores than females when adjusted for all variables, emphasising the importance of adjusted analyses in population studies. This higher depression score contradicted our hypothesis; one plausible explanation could be that males seek less primary care contact than females [24] as was the case in this cohort( Table 1, additional file). Our sub-analysis on gender and educational differences did not reveal any clear explanation, and the trend remained towards men scoring higher.

In line with prior studies, being married/cohabiting [9, 14] and higher education is associated with better mental health [8, 14] and economic difficulties [8, 11, 13–15] and unemployment [9, 14, 16] predict lower mental health. It is alarming to see the declining mental health of the young [8, 25]and that those already taking psychiatric medication for various mental health disorders reported low mental health. The ever-increasing use of antidepressant medication in the Icelandic nation [26], warming the top seat of the OECD countries for over a decade in antidepressant use, is not helping enough**.** It is crucial to remember that medical treatment and cognitive behavioural therapy for depression are the only forms of treatment partially subsidised by the government. Our results demonstrate the importance of the government subsidising various treatments, as there is no one-size-fits-all therapy. As a recent systematic review, demonstrated, the COVID-19 pandemic has increased psychological distress in the general population, intensifying depression, anxiety, and stress symptoms [27, 28] so the post-COVID19 pandemic healthcare systems might need to adapt to an escalation in mental health problems [12, 27, 28].

### Strengths and limitations

The strength of this study is that the sample is large, representative of the Icelandic population and covers both urban and rural areas of the country.

Our prevalence estimates may be biased because not all eligible individuals completed the questionnaire (Fig. 1). It is unknown whether the non-responders had better or worse mental health and, thereby, over- or underestimating the prevalence estimates. However, tendencies show that the motivation to participate in surveys is least among the socially disadvantaged in the population [29]. Hence, in that case, our prevalence results are underestimated.

Regarding the results in Table 3, estimates of associations between variables are not as vulnerable to bias as prevalence estimates [30]. Using multinomial logistic regression allows adjustments for co-variates to identify the most prominent sociodemographic factors associated with poor mental health, which is a great strength as it deepens our understanding of this population.

The cross-sectional study design, showing associations between sociodemographic characteristics, is valuable, although one cannot assume that the correlations are causal. Our data may be subject to self-report bias, meaning participants might not fully understand the questions or provide incorrect answers to make a good impression.

### Future suggestions

It seems necessary to introduce more interventions addressing mental health in Iceland. Van Agtern et al. concluded in their meta-analysis that mindfulness-based interventions are the most effective interventions for improving well-being [31]. It could be feasible to integrate cost-effective, evidence-based mental health interventions like Mindfulness-based Cognitive therapy [32] or therapies involving positive psychology interventions to the standard care [31] and tackle mental health via a broader lifestyle approach, focusing on nutrition, exercise, sleep and social connection. Knowing that their youngest population struggles with mental health problems are of great value for nations. Awareness of the connections between education, economic problems and mental health disorders could be used to implement national mental health intervention programs and support education. Implementing interventions at earlier stages of life and educating the young on the importance of good mental health, thereby destigmatising mental health problems, could be a start. Giving additional attention to those already suffering from mental disorders is essential, and medication use only seems insufficient. This information is of particular importance, considering the post-COVID-19 mental health problems nations worldwide seem to be facing [12, 27, 28].

## Conclusions

We found that young Icelanders show more mental health problems than older Icelanders. Unadjusted data showed similar depression scores for males and females, but males scored higher on depression and stress than females when adjusted for sociodemographic factors. Educational level, a cohabiting partner and financial security seem protective factors.

## Supplementary Information

**Additional file 1.**

## Data Availability

The data supporting this study's findings are publicly available upon request from The Icelandic Directorate of Health. Please contact the first author, Svala Sigurðardóttir, svala.sigurdardottir@clin.au.dk, regarding access to the dataset and materials.

## Abbreviations

DASS-21 scale: Depression, Anxiety, and Stress scale—21
RRR: Relative risk ratio
YLDs: Years lived with disability

## References

[1] Steel Z, Marnane C, Iranpour C (2014). The global prevalence of common mental disorders: a systematic review and meta-analysis 1980–2013. Int J Epidemiol.

[2] Plana-Ripoll O, Pedersen CB, Agerbo E (2019). A comprehensive analysis of mortality-related health metrics associated with mental disorders: a nationwide, register-based cohort study. Lancet.

[3] Vigo D, Thornicroft G, Atun R (2016). Estimating the true global burden of mental illness. Lancet Psychiatry.

[4] James SL, Abate D, Abate KH (2018). Global, regional, and national incidence, prevalence, and years lived with disability for 354 diseases and injuries for 195 countries and territories, 1990–2017: a systematic analysis for the Global Burden of Disease Study 2017. The Lancet.

[5] Wittchen HU, Jacobi F, Rehm J (2011). The size and burden of mental disorders and other disorders of the brain in Europe 2010. Eur Neuropsychopharmacol.

[6] Plana-Ripoll O, Pedersen CB, Holtz Y (2019). Exploring comorbidity within mental disorders among a Danish national population. JAMA Psychiatry.

[7] Prior A, Vestergaard M, Larsen KK (2018). Association between perceived stress, multimorbidity and primary care health services: a Danish population-based cohort study. BMJ Open.

[8] Rosendahl H, Davidsen M, Møller SR (2022). Danskernes sundhed: Den Nationale Sundhedsprofil 2021.

[9] Prior A, Fenger-Gron M, Larsen KK (2016). The Association Between Perceived Stress and Mortality Among People With Multimorbidity: A Prospective Population-Based Cohort Study. Am J Epidemiol.

[10] Salk RH, Hyde JS, Abramson LY (2017). Gender differences in depression in representative national samples: Meta-analyses of diagnoses and symptoms. Psychol Bull.

[11] Hapke U, Maske U, Scheidt-Nave C, et al. Chronic stress among adults in Germany. Bundesgesundheitsblatt-Gesundheitsforschung-Gesundheitsschutz. 2013;56:749–754. 10.1007/s00103-013-1690-9. 10.1007/s00103-013-1690-9 23703494

[12] Ettman CK, Abdalla SM, Cohen GH (2020). Prevalence of Depression Symptoms in US Adults Before and During the COVID-19 Pandemic. JAMA Netw Open.

[13] Ribeiro WS, Bauer A, Andrade MCR (2017). Income inequality and mental illness-related morbidity and resilience: a systematic review and meta-analysis. The Lancet Psychiatry.

[14] Santini ZI, Stougaard S, Koyanagi A, et al. Predictors of high and low mental well-being and common mental disorders: findings from a Danish population-based study. Eur J Public Health 2020. 10.1093/eurpub/ckaa021 10.1093/eurpub/ckaa021 PMC7292340 32104898

[15] Woodward EN, Walsh JL, Senn TE (2018). Positive social interaction offsets impact of low socioeconomic status on stress. J Natl Med Assoc.

[16] Paul KI, Moser K (2009). Unemployment impairs mental health: Meta-analyses. J Vocat Behav.

[17] Stefansson JG, Lindal E (2009). The prevalence of mental disorders in the Greater-Reykjavik area. Laeknabladid.

[18] OECD (2021). Mental health.

[19] Sigbjornsdottir HB, Gudlaugsson, J. O. and Jonsson, S. H. . Heilsa og líðan Íslendinga 2017: Framkvæmdaskýrsla. [Health and Wellbeing of Icelanders 2017. Project Report]. 2018 ed. Reykjavik, Iceland: Directorate of Health, 2018.

[20] Antony MM, Bieling PJ, Cox BJ (1998). Psychometric properties of the 42-item and 21-item versions of the Depression Anxiety Stress Scales in clinical groups and a community sample. Psychol Assess.

[21] Lovibond PF, Lovibond SH (1995). The structure of negative emotional states: Comparison of the Depression Anxiety Stress Scales (DASS) with the Beck Depression and Anxiety Inventories. Behav Res Ther.

[22] Sinclair SJ, Siefert CJ, Slavin-Mulford JM (2012). Psychometric Evaluation and Normative Data for the Depression, Anxiety, and Stress Scales-21 (DASS-21) in a Nonclinical Sample of U.S. Adults. Eval Health Prof.

[23] Seidler ZE, Dawes AJ, Rice SM (2016). The role of masculinity in men's help-seeking for depression: a systematic review. Clin Psychol Rev.

[24] Juel K, Christensen K (2008). Are men seeking medical advice too late? Contacts to general practitioners and hospital admissions in Denmark 2005. J Public Health.

[25] Danielsdottir S, Gudlaugsson JO, Gudmundsodttir DGM, health of Icelandic adults in,  (2020). Líðan fullorðinna Íslendinga árið 2020. Icelandic Directorate Health Newsl Health Data (Fréttabréf landlæknis um heilbrigðisupplýsingar).

[26] OECD (2021). Health at a Glance 2021.

[27] Xiong J, Lipsitz O, Nasri F (2020). Impact of COVID-19 pandemic on mental health in the general population: a systematic review. J Affect Disord.

[28] Magnúsdóttir I, Lovik A, Unnarsdóttir AB (2022). Acute COVID-19 severity and mental health morbidity trajectories in patient populations of six nations: an observational study. The Lancet Public Health.

[29] Goodman A, Gatward R (2008). Who are we missing? Area deprivation and survey participation. Eur J Epidemiol.

[30] Juul S (2013). Epidemiology and evidence.

[31] van Agteren J, Iasiello M, Lo L (2021). A systematic review and meta-analysis of psychological interventions to improve mental wellbeing. Nat Hum Behav.

[32] Kuyken W, Warren FC, Taylor RS (2016). Efficacy of mindfulness-based cognitive therapy in prevention of depressive relapse: an individual patient data meta-analysis from randomized trials. JAMA Psychiat.

