# Supplementary material for: Mental health and sociodemographic characteristics among Icelanders, data from a cross-sectional study in Iceland
Source: BMC Psychiatry. 2023 Jan 12;23:30. doi: 10.1186/s12888-022-04504-y (PMC9835021; doi:10.1186/s12888-022-04504-y)
Supplement: Supplementary file 1 — Additional file 1. [file 12888_2022_4504_MOESM1_ESM.docx]

**Additional file 1**

**Supplemental methods**

Influences of sex and antidepressant medication on depression score

Our results on depression scores between the genders contradicted our hypothesis about women scoring higher than men. In Supplementary table 1, we show antidepressant medication status by gender. Depression score by gender and antidepressant medication status is shown in Supplementary table 2.

It revealed that twice as many women were currently on antidepressant medication than men.

A multinomial logistic regression subanalysis was performed as well to explore further the differences mentioned above.

The general trend remained, with men scoring higher than women on depression, in all sub-analyses.

We additionally performed a likelihood-ratio test to see if the antidepressant medication intake was an effect modifier for the gender effect on the depression score. The test did point in the direction that it (antidepressant medication) can influence the differences between the sexes ( LRCchi = 10.45, Prob > chi2 =    0.0335).

Influences of educational level on depression score

Gender differences in educational levels are shown in Supplementary table 3.

More women had higher education, but a more significant part had only basic education.

To further investigate if educational level affected depression score differences between sexes, we did a subgroup logistic regression analysis. Comparing men and women with medium and high depression scores revealed a more difference between the sexes in middle and high education than the base difference. This was not significant and provided no apparent explanation. The tendency was consistently towards men scoring higher on depression than women.

A likelihood-ratio test to see if educational levels are an effect modifier was performed as well. The test did not reveal an effect modification of educational level on depression score between the sexes ( LR chi2(4) = 3.18, Prob > chi2 = 0.5274). The trend remained the same as before, with men having a higher risk of higher depression scores than women.

So the difference between the genders could not be explained by different educational levels. Still, as mentioned above, men with higher educational levels have higher crude depression scores than women with high education (Supplementary table 1).

**Supplementary table 1.**

Antidepressant medication by gender (Health and wellbeing of Icelanders 2017)

| Gender | Currently | % | Previously | % | Never | % | Total |
| --- | --- | --- | --- | --- | --- | --- | --- |
| Female | 288 | 8.0 | 216 | 6.0 | 3106 | 86.0 | 3610 |
| Male | 131 | 4.4 | 140 | 4.7 | 2729 | 91.0 | 3000 |
| Total | 419 | 6.3 | 356 | 5.4 | 5835 | 88.3 | 6610 |

**Supplementary table 2.**

Depression score (normal, medium, high) by gender and use of antidepressiva (Health and wellbeing of Icelanders 2017)

|  | Currently on antidepressiva | | | | |  | |  |  |  |
| --- | --- | --- | --- | --- | --- | --- | --- | --- | --- | --- |
| Gender |  |  | |  |  | |  |  |  |  |
|  | Normal | | % | Medium | | % | | High | % | Total |
| Female | 106 | | 38.8 | 89 | | 32.6 | | 78 | 28.6 | 273 |
| Male | 39 | | 33.1 | 47 | | 39.8 | | 32 | 27.1 | 118 |
| Total | 145 | | 37.1 | 136 | | 34.8 | | 110 | 28.1 | 391 |
|  | Previously on antidepressiva | | | | |  | |  |  |  |
| Female | 121 | | 60.2 | 65 | | 32.3 | | 15 | 7.5 | 201 |
| Male | 65 | | 49.6 | 42 | | 32.1 | | 24 | 18.3 | 131 |
| Total | 186 | | 56.0 | 107 | | 32.2 | | 39 | 11.8 | 332 |
|  | Never on antidepressiva | | | | |  | |  |  |  |
| Female | 2381 | | 83.7 | 404 | | 14.2 | | 60 | 2.1 | 2845 |
| Male | 2111 | | 83.7 | 355 | | 14.1 | | 57 | 2.3 | 2523 |
| Total | 4492 | | 83.7 | 759 | | 14.1 | | 117 | 2.2 | 5368 |

**Supplementary table 3.**

Education by gender (Health and wellbeing of Icelanders 2017)

|  | Basic |  | Middle |  | University |  | Total |
| --- | --- | --- | --- | --- | --- | --- | --- |
|  |  |  |  |  |  |  |  |
| Gender | N | % | N | % | N | % | N |
| Female | 1332 | 37.1 | 1022 | 28.5 | 1233 | 34.4 | 3587 |
| Male | 710 | 23.7 | 1575 | 52.6 | 709 | 23.7 | 2994 |
| Total | 2042 | 31.0 | 2597 | 39.5 | 1942 | 29.5 | 6581 |

**Supplementary table 4.**

Distribution of DASS-21 score for all participants (Health and wellbeing of Icelanders 2017)

| DASS-21 Score all | N | % |
| --- | --- | --- |
| Depression | 6185 |  |
| Normal | 4889 | 79.05 |
| Mild | 550 | 8.89 |
| Moderate | 474 | 7.66 |
| Severe | 140 | 2.26 |
| Extremely severe | 132 | 2.13 |
| Anxiety | 6256 |  |
| Normal | 5479 | 87.58 |
| Mild | 257 | 4.11 |
| Moderate | 345 | 5.51 |
| Severe | 77 | 1.23 |
| Extremely severe | 98 | 1.57 |
| Stress | 6213 |  |
| Normal | 5576 | 89.75 |
| Mild | 291 | 4.68 |
| Moderate | 219 | 3.52 |
| Severe | 106 | 1.71 |
| Extremely severe | 21 | 0.34 |
|  |  |  |

**Supplementary table 5.**

Distribution of DASS-21 score by gender (Health and wellbeing of Icelanders 2017)

| Males | N | % |
| --- | --- | --- |
| Normal | 2247 | 79.79 |
| Mild | 252 | 8.95 |
| Moderate | 202 | 7.17 |
| Severe | 56 | 1.99 |
| Extremely severe | 59 | 2.1 |
|  |  |  |
| Females |  |  |
| Normal | 2638 | 78.54 |
| Mild | 294 | 8.75 |
| Moderate | 271 | 8.07 |
| Severe | 84 | 2.5 |
| Extremely severe | 72 | 2.14 |
